# Case study on risk evaluation of printed electronics using nanosilver ink

**DOI:** 10.1186/s40580-016-0065-y

**Published:** 2016-02-18

**Authors:** Ellen Kim, Ji Hyun Lee, Jin Kwon Kim, Gun Ho Lee, Kangho Ahn, Jung Duck Park, Il Je Yu

**Affiliations:** 1 Institute of Nanoproduct Safety Research, Hoseo University, 165, Sechul-ri, Baebang-myun, Asan, Chungnam 336-795 Korea; 2 Department of Mechanics, Hanyang University, Ansan, Korea; 3 College of Medicine, Chung-Ang University, Seoul, Korea

**Keywords:** Silver Nanoparticle, Particle Number Concentration, Scanning Mobility Particle Sizer, Condensation Particle Counter, AgNP Exposure

## Abstract

**Background:**

With the ever-increasing development of nanotechnology, our society is being surrounded by possible risks related to exposure to manufactured nanomaterials. The consumer market already includes many products that contain silver nanoparticles (AgNPs), including various household products, such as yoga mats, cutting boards, running shirts, and socks. There is a growing concern over the release of AgNPs in workplaces related to the manufacture and application of nanomaterials.

**Objective:**

This study investigated the release of AgNPs during the operation of a printed electronics printer.

**Methods:**

Using an exposure simulation chamber, a nanoparticle collector, scanning mobility particle sizer (SMPS), condensation particle counter (CPC), dust monitor, and mixed cellulose ester (MCE) filters are all connected to measure the AgNP exposure levels when operating a printed electronics printer.

**Results:**

A very small amount of AgNPs was released during the operation of the printed electronics printer, and the number of AgNPs inside the exposure simulation chamber was lower than that outside background. In addition, when evaluating the potential risks for consumers and workers using a margin of exposure (MOE) approach and target MOE of 1000, the operational results far exceeded the target MOE in this simulation study and in a previous workplace exposure study.

**Conclusion:**

The overall results indicate a no-risk concern level in the case of printed electronics using nanosilver ink.

## Background

Printed electronics technology is an emerging area, where existing printing technologies are used to enable the flexible, large-area, low-cost, and environment-friendly mass production of electronic devices, such as organic photovoltaics (OPVs), copper indium gallium diselenide solar cells CIGSs), organic light emitting diodes (OLEDs), radio-frequency identification (RFID), and flexible printed circuit boards (FPCBSs) [1]. As printed electronics involves the convergence of various technologies, such as printing, fine mechanics, electronics, and nanotechnology, this raises new concerns over health and safety [2]. Especially, printed electronic devices include conductive nanoparticles, nanotubes such as single-walled nanotubes (SWCNTs) and multi-walled nanotubes (MWCNTs), nanoplates such as graphene, and new chemicals with unidentified health and safety effects. These nanomaterials used in printed electronics have been known to cause health effects in in vitro cellular toxicity studies as well as in vivo animal toxicity studies. Since risk is function of hazard and exposure, estimating exposure is a critical step in evaluating risk. Workers and researchers related to printed electronics are particularly at risk of exposure to nanomaterials, such as silver nanoparticles, representing the major conductive nanoink used in printed electronics. Despite difficulties in obtaining permission to do exposure assessment, the current authors already conducted several exposure assessment studies to evaluate the silver nanoparticle exposure in printed electronics workplaces. The results found a minimal level of silver nanoparticle exposure that did not represent any health and safety concern [3]. Further exposure simulation studies would be useful to clarify potential exposure and risk to silver nanoparticles.

Therefore, this study used an exposure simulation chamber to measure the mass and number concentration of nanoparticles released when operating a printed electronics printer using nanosilver ink. The health risks were also evaluated using a margin of exposure based on the NOAEL from silver nanoparticle toxicity data.

## Methods

### Conductive printer ink containing silver nanoparticles

According to the information provided by the manufacturer, the conductive print ink consisted of 32 % silver nanoparticles (20–30 nm), 7 % dispersing agents, and 60 % solvents.

### Printed electronics printer

According to the information provided by the manufacturer, a FUJIFILM Dimatix Materials Printer (DMP-2831, Santa Clara, CA) allows the deposition of fluidic materials on an A4 substrate utilizing a disposable piezo inkjet cartridge. This printer can develop patterns over an area of about 200 × 300 mm and handle substrates up to 25 mm thick with an adjustable height. The temperature of the vacuum platen, which secures the substrate in place, can be adjusted up to 60 °C. The DMP-2831 offers a variety of patterns using a pattern editor program. The inkjet cartridge is a piezo-driven jetting device with an integrated reservoir and heater, usable ink capacity: up to 1.5 ml (user-fillable), material compatibility: water-based solvents and acidic or basic fluids, 16 nozzles with 254 µm spacing in a single row, and drop volume: 1 (DMC-11601) and 10 (DMC-11610) picoliters nominal.

### Exposure simulation chamber

**Fig. 1:**
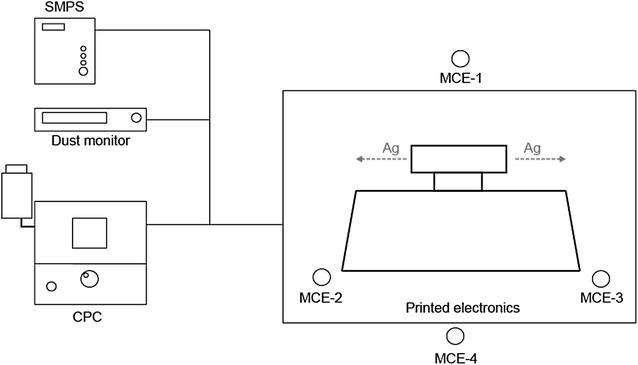
Schematic diagram of exposure simulation chamber

To estimate nanoparticle release quantitatively during operation of printed electronics, an exposure chamber was designed and exposure simulation studies were conducted using this chamber. The dimensions of the exposure simulation chamber were 1800 × 700 × 700 mm and it was made of acrylic, as shown in Fig. 1. To facilitate various measurements, such as the AgNP particle number, size distribution, and mass concentration, a SMPS, CPC, dust monitor, and MCE filters were all connected to the exposure simulation in which the DMP-2831 was placed and operated.

### Air sampling

Air samples taken by drawing air through MCE filters in sampling cassettes (0.45 μm, 37 mm support pad included) obtained from SKC Inc. were used for the total suspended particulate (TSP) measurements in terms of mass concentration, followed by a metal component analysis using an atomic absorption spectrometer (AAS). The filter samples for personal sampling were collected in the breathing zone using MSA (Escort Elf pump, Zefon International Inc. USA) sampling pumps operated at a flow rate of 1.92–2.07 L/min.

### Metal analysis

To estimate metal concentration, especially silver, air samples taken to MCE filters were analyzed. After wet digestion, the filter concentrations of residual metals were analyzed using an AAS equipped with a Zeeman graphite furnace (GF, pinAAcle 900T, Perkin Elmer, Waltham, MA) based on National Institute for Occupational Safety and Health (NIOSH) Manual method 7302 [4]. The filters were digested in a microwave (CEM MARS Xpress, Matthews, NC) for 60 min at 120 °C in the presence of a nitric acid to perchloric acid ratio of 4:1. Thereafter, the samples were allowed to cool and analyzed using AAS/GF. The limit of detection (LOD) and limit of quantitation (LOQ) for the Ag analysis using AAS were 0.098 and 0.323 ppb, respectively.

### Transmission electron microscopy (TEM)

TEM, including an energy dispersive X-ray analyzer (TEM-EDS), was used to measure the nanoparticles based on NIOSH analytical method 7402 [5]. The nanoparticles on the filter were mounted on a TEM grid (copper grid) and visualized under a field emission transmission electron microscope (FE-TEM, JEM2100F, JEOL, Japan). The nanoparticles were then measured at a magnification of 100,000 and analyzed using an EDS (TM200, Oxford, UK) at an accelerating voltage of 75 kV.

### Real-time aerosol monitoring

To estimate particle number concentration and particle size distribution released during operation of printed electronics, several particle analyzers were used. A scanning mobility particle sizer (SMPS), combining a differential mobility analyzer (DMA, 4220, HCT Co., Ltd, Icheon, Korea) and condensation particle counter (CPC, 4312, HCT Co., Ltd), was used to monitor the particle size distribution with an electrical mobility diameter ranging from 7.37 to 289.03 nm. Another condensation particle counter (CPC 3775, TSI Co., Ltd, 1–10^7^ particles/cm^3^, detection range) was used to monitor the number concentration. Plus, a dust monitor (Model 1.109, Grimm) was used to observe the particle size distribution with a diameter ranging from 0.25 to 32 µm. After starting the printed electronics printer, measurements were taken outside the exposure simulation chamber for 2 h and inside the exposure simulation chamber for 1 h. In addition, to measure the background, further measurements were taken outside the exposure simulation chamber for 2 h after terminating the printing operation.

### Risk analysis

A margin of exposure (MOE) approach was used to estimate the risk, where the calculated MOE was compared to a target MOE. Thus, if the calculated MOE is less than the target MOE, this represents a risk concern level, whereas if the calculated MOE is greater than the target MOE, this represents a no-risk concern level. In this study, the target MOE was set at 1000. The MOE_calc_ = POD/dose, where the POD is the toxicological point of departure according to the estimated dose to which humans will be exposed. In this study, the POD was 133 μg/m^3^ based on the NOAEL from a laboratory AgNP subchronic animal inhalation study [6].

## Results

### TEM analysis of nanosilver ink

**Fig. 2:**
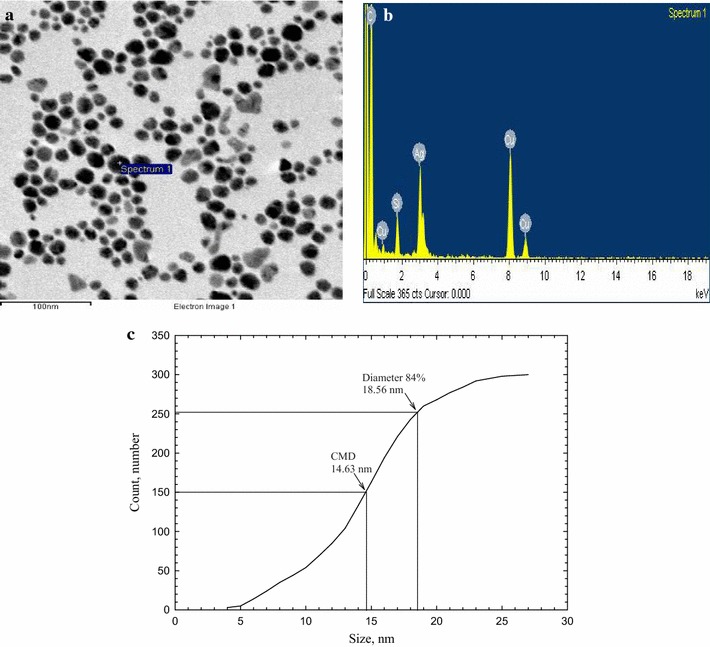
TEM-EDS (transmission electron microscopy) analysis of nanosilver ink. **a** TEM picture; **b** EDS; **c** count median diameter of silver nanoparticles

The silver nanoparticles contained in the nanosilver ink analyzed by TEM-EDX are shown in Fig. 2a. Most silver nanoparticles ranged from 4–28 nm with a count median diameter of 14.63 nm and 1.27 geometric standard deviation (Fig. 2c).

### Inhalation exposure to silver nanoparticles

Based on triplicate experiments, the AgNP exposure assessment data resulting from the operation of the printed electronics printer is presented in Table 1. The silver nanoparticle concentrations ranged from 0.01–0.02 μg/m^3^, representing very low time-weighted averages (TWAs) when compared with the current occupational exposure limits (0.1 mg/m^3^ for silver dust) suggested by the American Conference of Governmental Industrial Hygienists.

**Table 1:** Inhalation exposure to silver nanoparticles (μg/m^3^)

Ag concentration						
	Sample no	Pump flow rate (L/min)	Sampling time (min)	Ag conc (μg/m^3^)	MOE	MOE^a^
1st day	Exp 1	1.98	180	0.02	7600	38,000
	Exp 2	2.00	180	0.02		
	Exp 3	1.92	180	0.02		
	Exp 4	2.00	180	0.01		
2nd day	Exp 1	1.94	180	0.01	13,300	66,500
	Exp 2	1.91	180	0.01		
	Exp 3	2.01	180	0.01		
	Exp 4	2.00	180	0.01		
3rd day	Exp 1	1.97	180	0.01	10,640	53,200
	Exp 2	1.93	180	0.01		
	Exp 3	2.07	180	0.01		
	Exp 4	2.01	180	0.02		
Work place^b^	Personal 1	0.953	123	0.00024	554,166	2,770,833

*MOE* NOAEL/Exposure concentration, *MOE* ^*a*^ with use of personal protective equipment, 80 % reduction in inhalation exposure, *NOAEL* 133 μg/m^3^ Sung et al. [6]

^b^Data obtained from Lee et al. [3]

### Particle size distribution and number concentration during operation of printed electronics printer

**Fig. 3:**
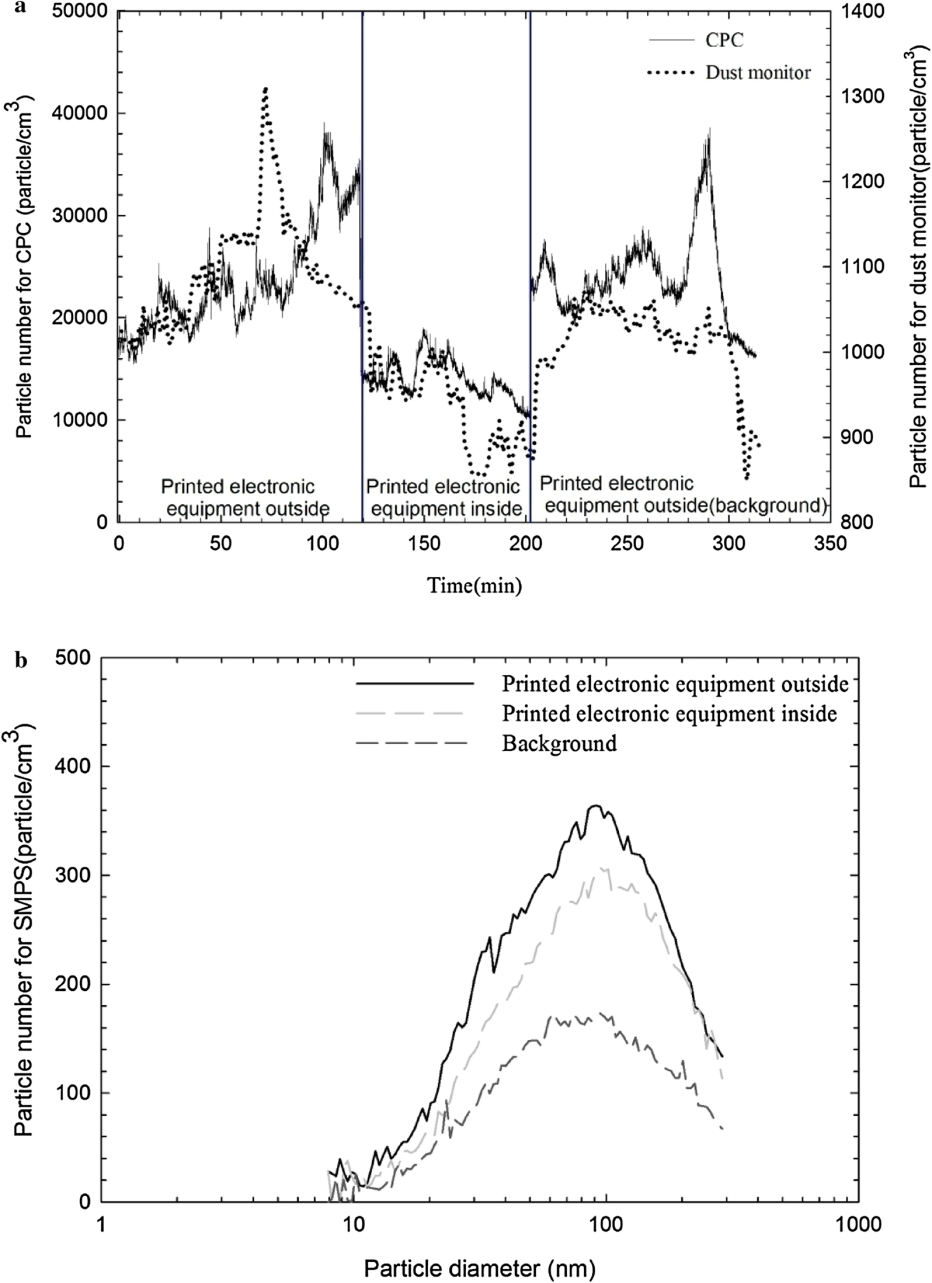
Particle distribution and number concentration during operation of printed electronics printer. **a** Measured using dust monitor and CPC; **b** measured using SMPS

The particle numbers (CPC) during the operation of the printed electronics printer were 13,710–39,130 particles/cm^3^ when measured outside the exposure simulation chamber and 9956–19,050 particles/cm^3^ when measured inside the exposure simulation chamber (Fig. 3a). During the operation of the printed electronics printer, an increase in particles larger than 0.25 μm was noted outside the exposure simulation chamber at 1004–1312 particles/cm^3^ compared to 852–1060 particles/cm^3^ inside the exposure simulation chamber (Fig. 3a). Meanwhile, the background (17:00–19:00) particle numbers were 14,084–38,620 particles/cm^3^ according to CPC and 850–1071 particles/cm^3^ according to the dust monitor.

Therefore, the particle number concentrations measured using CPC and the dust monitor showed higher exposure levels outside the exposure simulation chamber than inside when operating the printed electronics printer using nanosilver ink. Not withstanding, the number concentrations measured by SMPS showed very few nanoscale particles (less than 100 nm) at all the measurement sites. The size peaks for the particle numbers were all similar for exposure outside and inside during the operation of the printed electronics printer and exposure outside after the termination of the printed electronic printer (Fig. 3b).

### Risk evaluation of silver nanoparticle exposure during operation of printed electronics printer

The silver nanoparticle exposure risk was evaluated using an MOE approach. All the MOEs with or without personal protective equipment showed an MOE larger than 1000, set as the safe level (Table 1). The personal exposure levels previously measured at a printed electronics workplace by Lee et al. [3] also showed an MOE greater than 1000.

## Discussion

This simulation study of silver nanoparticle exposure when operating a printed electronics printer using nanosilver conductive ink is very useful for estimating silver nanoparticle exposure in the workplace. The exposure simulation chamber was connected to a CPC to count the particle number, SMPS to measure the size and count the nanoparticles, and dust monitor to measure and count the range of particles. The exposure simulation chamber also included a port for filter sampling to measure the mass concentration and for TEM and chemical analyses. As nanomaterials are an emerging technology, obtaining consent to conduct exposure assessment studies in workplaces that handle nanomaterials is a sensitive issue related to confidentiality. Therefore, the exposure simulation chamber used in this study offers a viable alternative environment for estimating workplace nanoparticle exposure without the hindrance of human and CBI elements.

Therefore, this study used the exposure simulation chamber to evaluate the silver nanoparticle exposure resulting from the operation of a printed electronics printer, and estimated the silver nanoparticle exposure risk using a margin of exposure approach. The results showed a very low silver nanoparticle exposure level during the operation of the printed electronics printer, plus the MOE was greater than 1000, representing a no-risk concern level. When compared with personal exposure levels measured at a printed electronics workplace, the MOE was also greater than 1000, supporting the data from the exposure simulation chamber. The low concentration of silver nanoparticles released was likely due to the high viscosity of the conductive ink containing silver nanoparticles.

The margin of exposure used in this study to estimate the exposure risk is commonly used by the US EPA to analyze the exposure risk for human health. In fact, the NOAEL or POD (point of departure) recommended by the US EPA and used in this study was originally obtained from a 90-day silver nanoparticle (18–19 nm) subchronic study [6]. Plus, following the FIFRA Scientific Advisory Panel meeting in 2009, the US EPA announced a conditional registration for the pesticide product HeiQ Material Ag (HeiQ) containing nanosilver as an active ingredient. In this case, the US EPA used a margin of exposure approach and data including the product chemistry, environment fate and effects, human exposure, and toxicology to assess the risks of consumer and worker exposure to HeiQ [7]. Thus, a similar approach was applied in this study to estimate the risk of exposure to silver nanoparticles potentially released during the operation of a printed electronics printer.

## Conclusion

A very small amount of AgNPs was released during the operation of the printed electronics printer. The number of AgNPs inside the exposure simulation chamber was lower than that outside background particle number. In addition, when evaluating the potential risks for consumers and workers using a margin of exposure (MOE) approach and target MOE of 1000, the operational results far exceeded the target MOE in this simulation study and in a previous workplace exposure study. Therefore, the overall results indicate a no-risk concern level in the case of printed electronics using nanosilver ink.
